# Biochemical and structural insights into a 5’ to 3’ RNA ligase reveal a potential role in tRNA ligation

**DOI:** 10.1073/pnas.2408249121

**Published:** 2024-10-10

**Authors:** Yingjie Hu, Victor A. Lopez, Hengyi Xu, James P. Pfister, Bing Song, Kelly A. Servage, Masahiro Sakurai, Benjamin T. Jones, Joshua T. Mendell, Tao Wang, Jun Wu, Alan M. Lambowitz, Diana R. Tomchick, Krzysztof Pawłowski, Vincent S. Tagliabracci

**Affiliations:** ^a^Department of Molecular Biology, University of Texas Southwestern Medical Center, Dallas, TX 75390; ^b^Department of Molecular Biosciences, University of Texas at Austin, Austin, TX 78712; ^c^Department of Oncology, University of Texas at Austin, Austin, TX 78712; ^d^Quantitative Biomedical Research Center, Peter O’Donnell Jr. School of Public Health, UT Southwestern Medical Center, Dallas, TX 75390; ^e^HHMI, University of Texas Southwestern Medical Center, Dallas, TX 75390; ^f^Harold C. Simmons Comprehensive Cancer Center, University of Texas Southwestern Medical Center, Dallas, TX 75390; ^g^Hamon Center for Regenerative Science and Medicine, University of Texas Southwestern Medical Center, Dallas, TX 75390; ^h^Green Center for Reproductive Biology Sciences, Department of Obstetrics and Gynecology, Children’s Research Institute, University of Texas Southwestern Medical Center, Dallas, TX 75390; ^i^Department of Biophysics, University of Texas Southwestern Medical Center, Dallas, TX 75390; ^j^Department of Biochemistry, University of Texas Southwestern Medical Center, Dallas, TX 75390

**Keywords:** C12orf29, RNA ligase, tRNA, RLIG1

## Abstract

ATP-grasp superfamily enzymes contain a hand-like ATP-binding fold and catalyze a variety of reactions using a similar catalytic mechanism. More than 30 protein families are categorized in this superfamily, and they are involved in a plethora of cellular processes and human diseases. Here, we identify C12orf29 (RLIG1) as an atypical ATP-grasp enzyme that ligates RNA. Human RLIG1 and its homologs autoadenylate on an active site Lys residue as part of a reaction intermediate that specifically ligates RNA halves containing a 5’-phosphate and a 3’-hydroxyl. RLIG1 binds tRNA in cells and can ligate tRNA within the anticodon loop in vitro. Transcriptomic analyses of *Rlig1* knockout mice revealed significant alterations in global tRNA levels in the brains of female mice, but not in those of male mice. Furthermore, crystal structures of a RLIG1 homolog from *Yasminevirus* bound to nucleotides revealed a minimal and atypical RNA ligase fold with a conserved active site architecture that participates in catalysis. Collectively, our results identify RLIG1 as an RNA ligase and suggest its involvement in tRNA biology.

## Significance

ATP-grasp enzymes share an atypical ATP-binding fold and catalyze a diverse set of reactions involved in many essential cellular processes. We identified C12orf29 (RLIG1) as an atypical ATP-grasp enzyme. Our biochemical and structural characterizations revealed this enzyme to be a 5’ to 3’ RNA ligase, structurally and functionally similar to phage T4 RNA ligase. RLIG1 can ligate tRNAs in vitro, and Rlig1 knockout female mice show altered tRNA levels in the brain. We also present structures of RLIG1, which reveal the mode of ATP binding and catalysis. Our work suggests that RLIG1 may play a pivotal role in the regulation of tRNAs.

ATP is widely used as an energy source by a variety of enzymes, including those with the ATP-grasp domain. These proteins share an ATP-binding fold characterized by two α + β subdomains that form a hand-like active site, allowing them to “grasp” an ATP molecule between two β-sheets. (1). Most ATP-grasp enzymes utilize an acceptor, such as a carboxylic acid, to form high-energy intermediates, which include acyl adenylates or acyl phosphates. These unstable intermediates are then attacked by a nucleophile, releasing the phosphate and forming a covalent bond between the carbon and the nucleophile (1). Although the reaction mechanism is generally conserved, the identities of nucleophiles and acceptors are diverse; thus, these enzymes differ in their biological functions. Some examples include biotin carboxylases involved in fatty acid synthesis (2), enzymes in the purine biosynthetic pathway (3), and nonribosomal peptide ligases (4).

Since their discovery in the 1990s (2), the ATP-grasp superfamily has expanded to more than 30 members (1, 5, 6), including the T4 phage 5’ to 3’ RNA ligase (T4 RNA ligase1, T4 Rnl1) (7). Although the 5’ to 3’ RNA ligases contain the ATP-grasp fold, their catalytic mechanism is different from canonical members of this superfamily; they transfer an AMP moiety from ATP to the side-chain amino group of an active-site lysine to form a ligase-(lysyl-N)-AMP intermediate. Subsequently, the AMP is transferred to the 5’phosphate of an RNA substrate to generate a high-energy phosphoanhydride intermediate. This intermediate is then attacked by the 3’ hydroxyl group of RNA to complete the ligation (8).

It is believed that there are more ATP-grasp enzymes yet to be discovered (1). We have previously taken a bioinformatics approach to identify new members of the protein kinase superfamily (9–13). Inspired by the diverse biological function and enzymatic activities of the ATP-grasp enzymes, we aimed to identify and characterize new family members using a similar approach to our work with protein kinases (14). This bioinformatic query identified the C12orf29 (RLIG1) protein as an atypical member of the ATP-grasp superfamily, which at the time was uncharacterized. Recently, RLIG1 was shown to be the first human 5’ to 3’ RNA ligase (15). However, the substrates and structural basis for catalysis remain unclear. Here, we show that the RLIG1 family of enzymes are 5’ to 3’ RNA ligases, which interact with tRNA in cells and specifically ligate the 5’ ends of the tRNA anticodon loop in vitro. Furthermore, *Rlig1* knockout female mice have alterations of tRNA levels in the brain. We resolve the structure of a member of this RNA ligase family, which reveals insights into catalysis. Our results describe a 5’ to 3’ RNA ligase potentially involved in tRNA biology.

## Results

### RLIG1 Is a 5’ to 3’ RNA Ligase.

**Fig. 1.**
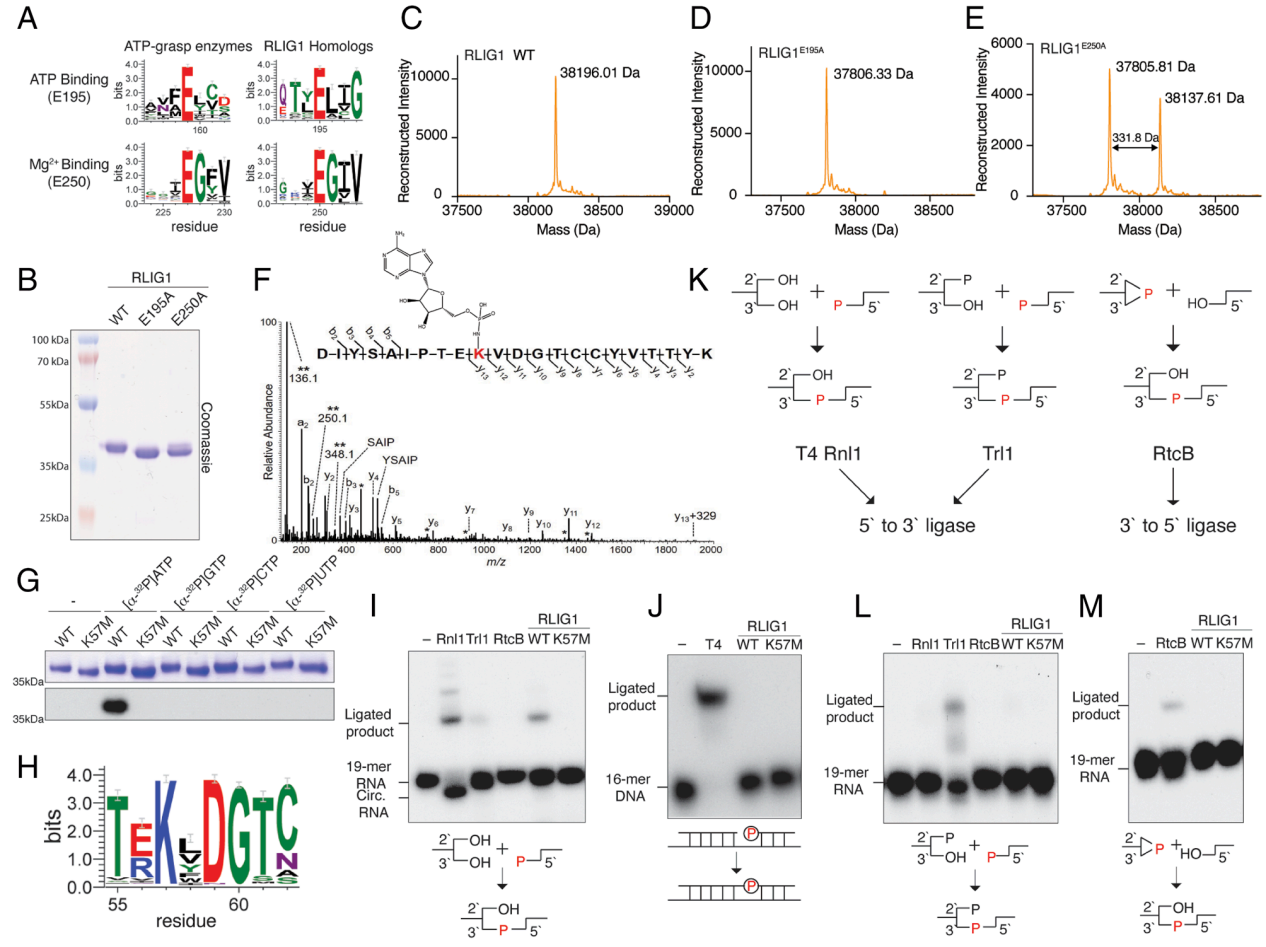
RLIG1 is a 5’ to 3’ RNA ligase. (*A*) Sequence logos highlighting conserved active site residues in 395 RLIG1 homologs (numbering as in human RLIG1) and 315 homologs of ATP-grasp-like enzymes (Pfam domain PF09511, rp15 dataset). The height of the amino acid stack is proportional to the sequence conservation at that position. Residues in parentheses are predicted human RLIG1 active site residues. (*B*) SDS-PAGE and Coomassie blue staining of recombinant RLIG1, RLIG1^E195A^, and RLIG1^E250A^. (*C*–*E*) Intact mass spectrum of WT RLIG1 (*C*) RLIG1^E195A^ (*D*) and RLIG1^E250A^ (*E*). The observed molecular weights are shown. A mass shift of 331.64 Da was observed between WT RLIG1 and RLIG1^E195A^. Note the similar mass shift between the 2 species in the RLIG1^E250A^ spectrum. (*F*) MS/MS spectrum of the adenylated peptide of RLIG1. The modified lysine residue is highlighted in red with the structure of covalently linked AMP shown. Unique ions corresponding to neutral loss of the AMP group (labeled with **) are present at 136.1, 250.1, and 348.1 Da. Peaks labeled with a single asterisk (*) correspond to b- and y-fragment ions with neutral loss of water (−18 Da). (*G*) Autoradiograph (*Lower*) and Coomassie blue staining (*Upper*) depicting the transfer of [^32^P]-labeled nucleotide monophosphates from [α-^32^P]-labeled nucleotide triphosphates onto RLIG1 or RLIG1^K57M^. One microgram of recombinant protein was used in each reaction. (*H*) Sequence logo of 395 RLIG1 homologs highlighting the conserved KxDG motif. (*I*) Autoradiograph depicting the reaction products from in vitro 5’ to 3’ RNA ligation assays. A ^32^P-labeled 19-mer ssRNA with 5’-phosphate, 3’-hydroxyl, and 2’-hydroxyl groups was incubated with known RNA ligases or RLIG1. Ligated and circularized products are indicated. Rnl1, T4 RNA ligase 1; Trl1, yeast tRNA ligase 1-388 (RNA ligase domain); RtcB, *E. coli* RtcB. Two micrograms of enzyme was used in each reaction. Schematic of the ligation is shown below. The radiolabeled phosphate group (P) is in red and incorporated into the newly formed phosphodiester bond. (*J*) Autoradiograph depicting the reaction products of in vitro nicked DNA ligation assays. A ^32^P-labeled 16-mer DNA and a nonlabeled 16-mer DNA were annealed to a 36-mer DNA to form a nicked DNA substrate. Nicked DNA was incubated with T4 DNA ligase (T4) or RLIG1. Two micrograms of enzyme was used in each reaction. Ligated products are indicated. Schematic of the nicked DNA ligation is shown below. The radiolabeled phosphate group (P) is shown in red. (*K*) Schematics depicting the 5’ to 3’ and the 3’ to 5’ RNA ligation pathways carried out by the 5’ to 3’ RNA ligases T4 Rnl1 and yeast Trl1 and the 3’ to 5’ RNA ligase RtcB. The phosphate in the newly formed phosphodiester bond originating from either the 5’ or the 3’ end of the RNA is shown in red. (*L*) Autoradiograph depicting the reaction products of in vitro 5’ to 3’ RNA ligation assays. A ^32^P-labeled 19-mer ssRNA with 5’-phosphate, 3’-hydroxyl, and 2’-phosphate groups was incubated with known RNA ligases, Rnl1, Trl1, RtcB, or RLIG1. Two micrograms of enzyme was used in each reaction. Schematic of the ligation is shown below. The phosphate incorporated into the newly formed phosphodiester bond is shown in red. (*M*) Autoradiograph depicting the reaction products of in vitro 3’ to 5’ RNA ligation assays. A ^32^P-labeled 19-mer ssRNA with 5’ hydroxyl, 2’,3’-cyclic phosphate groups was incubated with RtcB or RLIG1. Two micrograms of enzyme was used in each reaction. Schematic of the ligation is shown below. The phosphate incorporated into the newly formed phosphodiester bond is shown in red.

Sensitive sequence searches using the fold and function assignment system (FFAS) identified the C12orf29 (RLIG1) family of proteins as atypical ATP-grasp enzymes (16). FFAS alignments to known structures and sequence logo analyses (17) of RLIG1 homologs suggested two residues corresponding to the ATP-binding glutamate (E195) and Mg^2+^ binding glutamate (E250) of the prototypical ATP-grasp enzymes (1) (Fig. 1*A*). To gain insight into the enzymatic activity of RLIG1, we expressed and purified the recombinant human protein and two predicted active site mutants (E195A and E250A) in *Escherichia coli*. SDS-PAGE analysis revealed a slight mobility shift between the WT and the E195A protein (Fig. 1*B*). Moreover, two bands were present in the E250A protein with somewhat similar electrophoretic mobilities as the WT and the E195A mutant. Intact mass spectrometry analysis revealed a 331.64 Da mass shift between the WT and E195A protein, roughly corresponding to the addition of a single AMP molecule (Fig. 1 *C* and *D*). We also observed two peaks separated by ~331.8 Da in the spectrum of the E250A protein (Fig. 1*E*). LC-MS/MS confirmed that a single AMP molecule was covalently linked to K57 (Fig. 1*F*). We incubated the WT and a K57M mutant of RLIG1 with [α-^32^P]-ATP and observed ^32^P incorporation into the WT protein but not the K57M mutant (Fig. 1*G*). The reaction was specific for ATP, as RLIG1 was unable to incorporate ^32^P from the other [α-^32^P]-labeled nucleotide triphosphates tested. Collectively, these data suggest that human RLIG1 catalyzes the autoadenylation of K57 in vitro.

The adenylated lysine in RLIG1 lies within a conserved KxDG motif (Fig. 1*H*), which is reminiscent of the ATP-dependent polynucleotide ligases whose reaction proceeds through a ligase-(lysyl-N)-AMP intermediate (8). To determine whether RLIG1 is a polynucleotide ligase, we performed in vitro ligation assays using ^32^P-radiolabeled nicked DNA or a ^32^P-radiolabeled single-stranded 19-mer RNA with a 5’-phosphate and a 3’-hydroxyl. RLIG1 ligated single-stranded RNA (ssRNA) (Fig. 1*I*) but not nicked DNA (Fig. 1*J*), suggesting that RLIG1 is an RNA ligase. Interestingly, while the 19-mer ssRNA mostly circularized with some oligomer formation when incubated with T4 Rnl1, RLIG1 specifically catalyzed the formation of the dimer species. (Fig. 1*I*). RNA ligases catalyze the formation of the 5’ to 3’phosphodiester bond and are categorized by their substrate specificity (Fig. 1*K*) (18). T4 RNA ligase 1 (T4 Rnl1) exemplifies RNA ligases that require a 5’-phosphate, 3’-hydroxyl, and 2’-hydroxyl group (19). The yeast tRNA ligase, Trl1, ligates RNA with a 5’-phosphate, 3’-hydroxyl, and 2’-phosphate (20). These two types of RNA ligases are collectively referred to as 5’ to 3’ RNA ligases because the phosphate in the newly formed phosphodiester bond originates from the 5’ end (18). The third kind of RNA ligase, RtcB, joins a 2’,3’-cyclic phosphate and a 5’-hydroxyl group (21). RtcB is a 3’ to 5’ ligase because the phosphate originates from the 3’ end. To test the substrate specificity of RLIG1, we performed in vitro ligation assays using radiolabeled ssRNA with a 5’-phosphate, 3’-hydroxyl, and 2’-phosphate group (Fig. 1*L*) or with a 2’,3’-cyclic phosphate and a 5’-hydroxyl group (Fig. 1*M*). Neither substrate was ligated by RLIG1, whereas yeast Trl1 and *E. coli* RtcB ligated their respective RNA species. Thus, RLIG1 is a 5’ to 3’ RNA ligase that joins RNA halves containing a 5’-phosphate, and a 2’,3’-hydroxyl in vitro. Our results confirm and complement the recent study by Yuan et al. (15).

### tRNA Is a Potential Substrate of RLIG1.

**Fig. 2.**
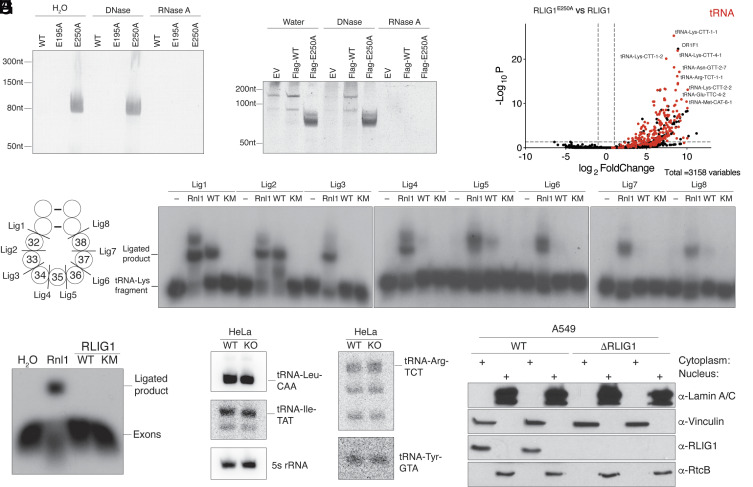
tRNA is a potential substrate of RLIG1. (*A*) Urea-PAGE and SYBR Gold staining depicting the nucleic acids that copurified with recombinant RLIG1 or mutants from *E. coli*. Samples were treated with DNase or RNase A and digested with proteinase K prior to analysis. (*B*) Urea-PAGE and SYBR Gold staining depicting the nucleic acids that copurified with α-Flag-immunoprecipitates from HEK293A cells expressing Flag-tagged RLIG1 or RLIG1^E250A^. Immunoprecipitates were treated with DNase or RNase A and digested with proteinase K prior to analysis. (*C*) Volcano plot depicting the enrichment of tRNA in the α-Flag-immunoprecipitates from HEK293A cells expressing Flag-tagged RLIG1^E250A^ vs Flag-tagged RLIG1. RNAs were analyzed by AQ-seq. tRNA is labeled in red. The fold change cutoff is 2 and the *P*-value cutoff is 0.05. (*D*) Schematic depicting a segment of the tRNA anticodon stem-loop. Cleavage sites are shown, and the nucleotides are numbered according to the canonical tRNA nucleotide numbering system. For simplicity, only the anticodon loop of tRNA is shown. In the ligation assay, two RNA fragments are joined to form full-length tRNA. (*E*) Autoradiograph depicting the reaction products of an in vitro tRNA-Lys-CTT fragment ligation assay using T4 Rnl1 (Rnl1) and human RLIG1 (WT) or RLIG1^K57M^ (KM). A synthetic tRNA-Lys-CTT 3’ fragment was labeled with ^32^P at the 5’ end, annealed to an unlabeled 5’ fragment, and incubated with the ligases (2 μg). Substrates are indicated in (*D*). (*F*) Autoradiograph depicting an in vitro exon ligation assay using Rnl1 and human RLIG1 or RLIG1^K57M^. *Saccharomyces cerevisiae* pre-tRNA-Phe-GAA was transcribed in vitro and cleaved with the TSEN endonuclease complex. Exons were purified following Urea-PAGE electrophoresis and were processed and labeled with T4 Pnk using [γ-^32^P]ATP, after which they were incubated with the ligases (2 μg). (*G*) Northern blot depicting intron-containing tRNAs from WT or *RLIG1* KO Hela cells. 5S rRNA is shown as a loading control. (*H*) Protein immunoblots depicting Lamin A/C, Vinculin, RLIG1, and RtcB. A549 cell lysates were fractionated to separate the nucleus from the cytoplasm. Lamin A/C is used as a nuclear marker and Vinculin as a cytosolic marker.

During purification of RLIG1 from bacterial lysates, we observed that RLIG1^E250A^, unlike the WT or the RLIG1^E195A^, copurified with nucleic acids (Fig. 2*A* and *SI Appendix*, Fig. S1 *A* and *B*). Urea-PAGE analysis revealed a species of ~80nt that was resistant to DNase but not RNase treatment (Fig. 2*A*). Based on the size, we speculated that the RNA was tRNA. Indeed, Northern blot analysis detected *E. coli* tRNA-Ala-GGC (*SI Appendix*, Fig. S1*C*). Because RLIG1^E250A^ was partially adenylated (Fig. 1 *B* and *E*) and copurified with tRNA, we hypothesized that it may act as a substrate-trapping mutant. Therefore, to identify possible substrates in human cells, we generated stable HEK293A cell lines expressing Flag-tagged RLIG1 or RLIG1^E250A^ (*SI Appendix*, Fig. S1*D*). Urea-PAGE analysis of anti-Flag immunoprecipitates revealed an ~50 to 100nt long RNA species that was exclusively enriched in the RLIG1^E250A^ immunoprecipitates (Fig. 2*B*). To identify the RNA species, we excised a region of the Urea-PAGE gel between 50 to 200 nt and performed accurate quantification by sequencing (AQ-seq) (22). The top hits enriched in the Flag-RLIG1^E250A^ sample were predominantly tRNAs (Fig. 2*C* and *SI Appendix*, Fig. S1*E* and Datasets S1 and S2), which were confirmed by Northern blotting for tRNA-Lys-CTT, the most highly enriched tRNA (*SI Appendix*, Fig. S1*F*). tRNA undergoes cleavage at the anticodon loop as part of intron splicing or in response to stress by a variety of nucleases (18, 23, 24). To test whether RLIG1 can ligate tRNA containing a 5’phosphate and a 3’ hydroxyl in vitro, we prepared tRNA-Lys-CTT fragments corresponding to the full-length tRNA cleaved at each position of the anticodon loop (Fig. 2*D*) and performed tRNA ligation assays. Interestingly, RLIG1 preferred to ligate fragments excised between nucleotides 31 to 32 (Lig1) and 32 to 33 (Lig2) of the anticodon loop (Fig. 2*E*). Although RLIG1 can catalyze the ligation of Lig1 and Lig2, the specific activity of RLIG1 for Lig2 was an order of magnitude higher than for Lig1; however, the reactions were quite slow (*SI Appendix*, Fig. S1 *G* and *H*).

About 6% of human tRNAs contain an intron that is cleaved in the nucleus by the tRNA splicing endonuclease (TSEN) complex generating tRNA halves with a 5’ hydroxyl and a 2’,3’ cyclic phosphate (18). The exons are then ligated by the nuclear 3’ to 5’ RNA ligase RtcB, which is considered the major tRNA splicing ligase (21). Interestingly, a yeast Trl1-like 5’ to 3’ tRNA splicing ligase activity was detected in human nuclear cell lysates over 30 y ago (25). To test whether RLIG1 is a tRNA splicing ligase, we in vitro transcribed yeast pre-tRNA-Phe-GAA, cleaved the intron with the TSEN complex, then purified and processed the exons with T4 polynucleotide kinase (Pnk) to generate halves with a 5’-phosphate and a 3’-hydroxyl. While T4 Rnl1 efficiently ligated the tRNA exons, RLIG1 did not (Fig. 2*F*). Likewise, we generated *RLIG1* KO HeLa cells (*SI Appendix*, Fig. S1*I*) and probed for intron-containing tRNAs; however, we did not observe any differences in the human intron-containing tRNAs (Fig. 2*G*). Furthermore, RLIG1 predominantly localized to the cytoplasm (Fig. 2*H*), while tRNA splicing occurs in the nucleus (26). Thus, although tRNA is a potential substrate of RLIG1, it does not appear to be a splicing tRNA ligase.

### Female *Rlig1* KO Mice have Alterations in Small Noncoding Transcripts.

**Fig. 3.**
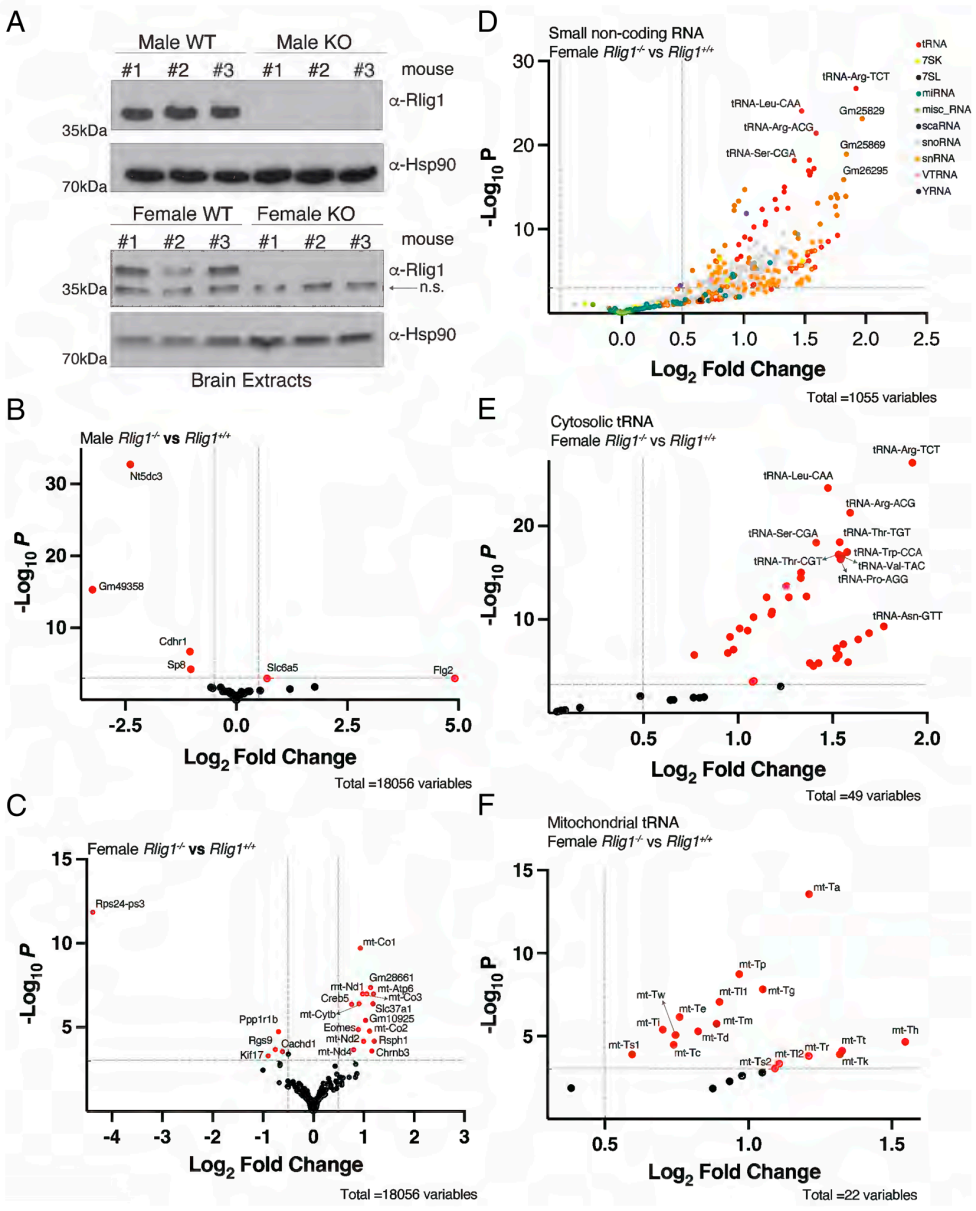
Female *Rlig1* KO mice have alterations in small noncoding transcripts. (*A*) Protein immunoblots of mouse brain lysates depicting Rlig1 levels in *Rlig1*^+/+^ and *Rlig1*^−/−^ mice. Hsp90 is shown as a loading control. n.s. stands for nonspecific. (*B* and *C*) Volcano plot from TGIRT-seq analysis comparing protein-coding genes or pseudogenes from male (*B*) or female (*C*) *Rlig1*^−/−^ and *Rlig*1^+/+^ mice. Genes significantly changed are in red. The log_2_ fold change cutoff is 0.5, and the *P*-value cutoff is 0.001. (*D*) Volcano plot from TGIRT-seq analysis comparing small noncoding transcripts from female *Rlig1*^−/−^ and *Rlig1*^+/+^ mice. The log_2_ fold change cutoff is 0.5, and the *P*-value cutoff is 0.001. (*E* and *F*) Volcano plot from TGIRT-seq analysis comparing cytosolic tRNAs (*E*) or mitochondrial tRNAs (*F*) from female *Rlig1*^−/−^ and *Rlig1*^+/+^ mice. tRNAs significantly changed are in red. The log_2_ fold change cutoff is 0.5, and the *P*-value cutoff is 0.001.

We produced *Rlig1* knockout mice (*Rlig1^−/−^)* in an ICR/CD1 background using CRISPR-Cas9 technology with two guide RNAs targeting exon 2 of the mouse gene (Fig. 3*A* and *SI Appendix*, Fig. S2 *A* and *B*). Intercrosses of *Rlig1^+/−^* mice produced all genotypes at the expected Mendelian frequencies (*SI Appendix*, Fig. S2*C*). Although two *Rlig1*^−/−^ mice from the original litter displayed seizures, we were unable to reproduce this phenotype in subsequent cohorts. As such, no overt phenotypes were observed upon deletion of *Rlig1* in mice.

We extracted total RNA from brains and profiled the transcriptome using TGIRT-seq (27). There were only 2 protein-coding genes or pseudogenes up-regulated and 4 down-regulated in male KO mice; 15 genes were up-regulated and 5 down-regulated in female KO mice (Fig. 3 *B* and *C* and Datasets S3 and S4), suggesting that *Rlig1* KO has little effect on the expression of protein-coding genes or pseudogenes. Only a few long noncoding genes were significantly changed upon *Rlig1* deletion in female mice and none of them in male KO mice (*SI Appendix*, Fig. S2 *D* and *E* and Datasets S5 and S6), suggesting that *Rlig1* does not significantly influence the expression of long noncoding RNAs. Interestingly, *Rlig1* deletion led to upregulation of many small noncoding RNAs in female KO mice. Most of the up-regulated small noncoding RNAs were tRNAs, small nucleolar RNAs (snoRNAs), and small nuclear RNAs (snRNAs) (Fig. 3*D* and Dataset S6). Surprisingly, most cytosolic tRNAs and mitochondrial tRNAs were up-regulated in female KO mice (Fig. 3 *E* and *F* and Dataset S6). However, these trends were not observed in male KO mice (*SI Appendix*, Fig. S2*F* and Dataset S5). tRNAs are transcribed by RNA polymerase III (Pol III), prompting us to investigate whether the upregulation of tRNAs in female *Rlig1*^−/−^ mice was due to increased Pol III activity. However, we did not observe any upregulation of other Pol III transcripts in female KO mice (*SI Appendix*, Fig. S2*G* and Dataset S7). Because RLIG1 binds to tRNA and ligates the anticodon loop, we tested tRNA fragment levels in WT and *Rlig1*^−/−^ mice; however, we observed no differences (*SI Appendix*, Fig. S2 *H* and *I*). Thus, *Rlig1*^−/−^ mice are viable, display no overt phenotypes, and females have elevated levels of small noncoding transcripts in the brain. Future research is necessary to unravel the underlying sex-specific mechanisms responsible for the upregulation of small noncoding RNAs in *Rlig1^−/−^* female mice.

### Yasminevirus RLIG1 Adopts an ATP-grasp RNA Ligase Fold.

**Fig. 4.**
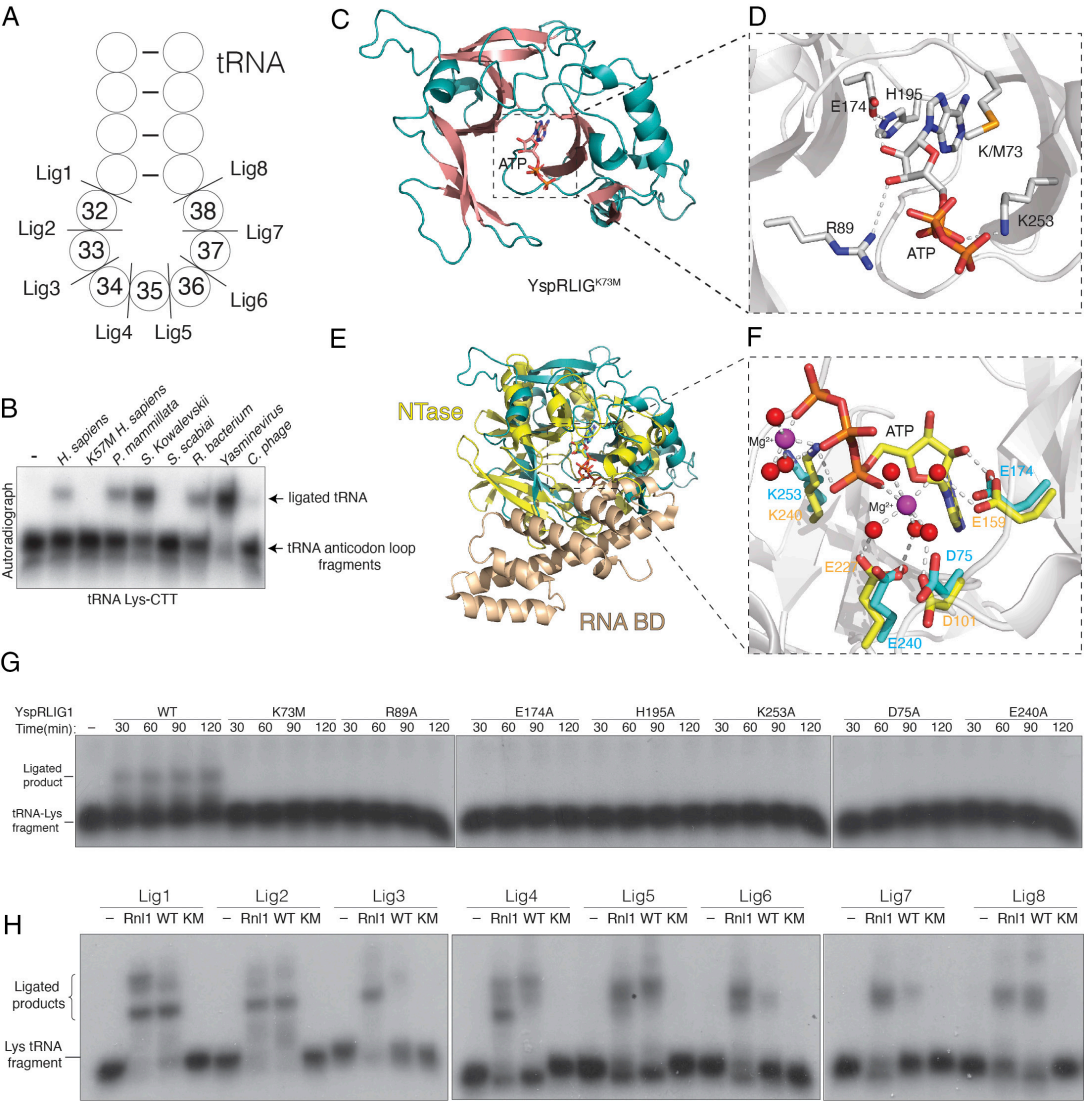
Yasminevirus RLIG1 adopts an ATP-grasp RNA ligase fold. (*A*) Schematic depicting a segment of the Lys-tRNA anticodon stem-loop. Cleavage sites are shown, and the nucleotides are numbered according to the canonical tRNA nucleotide numbering system. For simplicity, only the anticodon loop of tRNA is shown. In the ligation assay, two RNA fragments are joined to form full-length tRNA. (*B*) Autoradiograph depicting the reaction products of an in vitro tRNA-Lys-CTT fragment ligation assay (nt. 32 to 33) using human RLIG1 and its homologs (2 μg). (*C*) Cartoon representation of YspRLIG1^K73M^. The α-helices and β-strands are colored in teal and salmon, respectively. The nucleotide is shown as sticks. (*D*) Zoomed-in view depicting the active site residues involved in coordinating nucleotide. Residues and ATP are shown as sticks. Interactions are denoted by dashed lines. (*E*) Superposition of YspRLIG1^K73M^ with T4 Rnl1 (PDB: 5tt6). YspRLIG1^K73M^ is in teal and the nucleotidyltransferase (NTase) and RNA binding domains (RNA BD) of T4 Rnl1 are in yellow and wheat, respectively (PDB: 5tt6). (*F*) Zoomed-in view depicting the active site residues of YspRLIG1^K73M^ and T4 Rnl1. Coloring as in (*E*). Magenta spheres denote magnesium ions, and red spheres denote water molecules. The ATP, Mg^2+^, and water molecules are from the T4 Rnl1 structure. (*G*) Autoradiograph depicting the time course reaction products of an in vitro tRNA-Lys-CTT fragment ligation (nt. 31 to 32) assay using YspRLIG1 or mutants. A synthetic tRNA-Lys-CTT 3’ fragment was labeled with ^32^P at the 5’end, annealed to an unlabeled 5’ fragment, and incubated with the ligases (2 μg). (*H*) Autoradiograph depicting the reaction products of an in vitro tRNA-Lys-CTT fragment ligation assay using T4 Rnl1 (Rnl1) and YspRLIG1 (WT) or YspRLIG1^K73M^ (KM). A synthetic tRNA-Lys-CTT 3’ fragment was labeled with ^32^P at the 5’end, annealed to an unlabeled 5’ fragment, and incubated with the ligases (2 μg). Substrates are indicated in (*A*).

RLIG1 homologs are found in some bacteria, viruses, and many metazoans (*SI Appendix*, Fig. S3). To test whether RLIG1 homologs are active RNA ligases, we expressed and purified several bacterial and viral proteins and performed in vitro ligation assays using tRNA-Lys-CTT fragments corresponding to the full-length tRNA cleaved at the anticodon loop (Fig. 4*A*). Indeed, several homologs efficiently ligated the tRNA halves containing a 5’ phosphate and 3’ hydroxyl (Fig. 4*B*, nt. 32 to 33).

To gain insight into the reaction mechanism of RLIG1, we solved crystal structures of WT (adenylated) and K73M *Yasminevirus sp.* GU-2018 (28) RLIG1 (YspRLIG1) in the presence of ATP (Fig. 4*C* and *SI Appendix*, Fig. S4*A* and Table S1). In the WT structure, either AMP or ADP was modeled in the active site (*SI Appendix*, Fig. S4*A*). In the YspRLIG1^K73M^ structure, ATP was bound in the active site (Fig. 4 *C* and *D*). Both structures were virtually identical (*SI Appendix*, Fig. S4*B*); thus, we use the YspRLIG1^K73M^ structure to describe the interactions related to catalysis. YspRLIG1^K73M^ clamps the ATP molecule with two antiparallel β-sheets, a common feature of the ATP-grasp fold and the RNA ligase superfamily (Fig. 4 *C* and *D* and *SI Appendix*, Fig. S4*C*) (29). Structural homology searches using the Dali server (30) identified *Naegleria gruberi* RNA ligase (PDB: 5cot) (29) as the top hit, with all 10 significant hits being RNA or DNA ligases. We superimposed YspRLIG1^K73M^ with T4 Rnl1 (PDB: 5tt6) (31) and calculated a RMSD of 4.83 Å over 136 residues (Fig. 4 *E* and *F*). Although YspRLIG1 shares the nucleotidyltransferase (NTase) domain of T4 Rnl1, it is missing the RNA binding domain (31, 32).

The NTase domains of the polynucleotide ligases share conserved motifs (I, Ia, III, IIIa, IV, and V) that are involved in coordinating nucleotide and metal ion (8, 33). These motifs are also conserved in homologs of RLIG1 (*SI Appendix*, Fig. S3*A*). We superimposed the active sites of ADP-bound YspRLIG1^WT^ with ATP-bound YspRLIG1^K73M^ (*SI Appendix*, Fig. S4*D*). The pyrophosphate group of the ATP from the YspRLIG1^K73M^ structure is not poised for catalysis because the Lys73 (Motif I) is replaced with a Met and is 6.4 Å from the Pα of the ATP (Nζ-Pα-O3α angle = 85.4°). In the ADP-bound YspRLIG1^WT^ structure, the O5-Pα bond is rotated to flip the α-phosphate group toward the Nζ of Lys73. This situates the Nζ of Lys73 ~4Å away from the Pα of the ADP in an apical orientation to the β-phosphate group (Nζ-Pα-O3α angle = 144.4°). We believe this orientation more likely represents the conformation of the α-phosphate group during adenylation. Arg89 (Motif Ia) coordinates ATP-binding and forms a hydrogen bond with the 3’-OH of the ribose ring (Fig. 4*D*). Glu174 (Motif III), His195 (Motif IIIa), and the 2’-OH of the ribose ring form a hydrogen bonding network that precisely orients the nucleotide in the active site. Lys253 (Motif V) is involved in neutralizing the negative charge from the nucleotide (Fig. 4*D*). Mutations of these residues reduced autoadenylation (*SI Appendix*, Fig. S4*E*) and RNA ligation (Fig. 4*G*, nt. 31 to 32). Superposition of the YspRLIG1^K73M^ and T4 Rnl1 active sites suggests that Asp75 (Motif I), Glu174 (Motif III), Glu240 (Motif IV), and Lys253 (Motif V) may be involved in Mg^2+^ binding (Fig. 4*F*). We modeled a Mg^2+^ ion in the electron density that is between 3.5 to 4 Å from these residues, which is a density that would be consistent with a Mg(H_2_O)_6_^2+^ ion; however, we do not see the bound waters (*SI Appendix*, Fig. S4*F*). In any event, mutation of these residues and removal of divalent cations with EDTA markedly reduced adenylation activity (*SI Appendix*, Fig. S4*E*) and RNA ligation (Fig. 4*G*, nt. 31 to 32 and *SI Appendix*, Fig. S4*G*).

We performed in vitro YspRLIG1 ligation assays using tRNA-Lys-CTT fragments corresponding to the full-length tRNA cleaved at each position of the anticodon loop (Fig. 4*A*). When compared to the specificity of the human protein (Fig. 2*E*), YspRLIG1 displayed a broader substrate preference for the anticodon loop (Fig. 4*H*). Interestingly, when we superimposed the AlphaFold (34) model of human RLIG1 with YspRLIG1 (RMSD of 2.91 Å), YspRLIG1 is missing an N-terminal segment that is found in the human protein (aa. 81 to 111) (*SI Appendix*, Fig. S4*H*). However, deletion of this segment did not generate a more promiscuous RNA ligase (*SI Appendix*, Fig. S4*I*). Thus, specificity for RNA appears to be encoded in the active site and the N-terminal segment in human RLIG1 may have a regulatory function. Collectively, our structural analyses of YspRLIG1 reveal a minimal and atypical RNA ligase fold and highlight key active site residues involved in RNA ligation.

## Discussion

Our work uncovers RLIG1 as a 5’ to 3’ RNA ligase. Although we reported this activity in 2022 (35), we delayed publication because our initial cohort of *Rlig1*^−/−^ mice displayed a seizure phenotype; however, subsequent cohorts failed to reproduce this phenotype. During our characterization of the mice, Yuan et al. also identified RLIG1 by chemical proteomics as a 5’ to 3’ RNA ligase (15). Our work complements their discovery and adds tRNA as a potential substrate of RLIG1 in vitro and in vivo. Furthermore, we report structures of RLIG1, which have shed light on the catalytic mechanisms used by this family of RNA ligases.

5’ to 3’ RNA ligase was originally purified from T4 phage-infected bacteria (36), and the phage ligase was discovered as part of a tRNA repair mechanism which antagonizes host immunity 15 y later (19). As a phage defense response, some bacteria will induce a nuclease, PrrC, which cleaves its own tRNA-Lys and induces cell death to prevent phage replication (19, 37). To combat cell death, T4 phage encodes a polynucleotide kinase (Pnk) and an RNA ligase (Rnl1) to repair the cleaved tRNA. Pnk has two domains: a phosphatase domain, which removes the 2’,3’-cyclic phosphate, and an RNA kinase domain, which phosphorylates the 5’-hydroxyl generated by PrrC. Both ends are then joined by T4 Rnl1 (19, 37). It is worth noting that several viruses have a RLIG1 homolog (*SI Appendix*, Fig. S3), and we predict that viral RLIG1 may be used to combat host immunity similar to T4 Rnl1. Interestingly, all the enzymatic activities of the T4 repair system have now been identified in humans. A human 2’,3’-cyclic phosphodiesterase named ANGEL2 (38) and a 5’-hydroxyl RNA kinase named hClp1 (39) could serve as functional equivalents to Pnk. Likewise, RLIG1 is a functional homolog of Rnl1. It remains to be seen whether these activities are coordinated in human cells. Many human RNases generate a 3’-phosphate (18, 35, 40), which would require modification prior to ligation by RLIG1. We postulate that humans possess a similar tRNA repair mechanism to T4 phage.

Yasminevirus is a member of the *Klosneuvirinae* group of giant viruses and was isolated from a coculture with the amoeba *Vermamoeba vermiformis*. Its genome spans 2.1 Mb, encoding an almost complete translational machinery that includes 70 tRNAs, 20 aminoacyl-tRNA synthetases, and several translation and elongation factors (28). Interestingly, it also encodes for a protein (Uniprot entry: A0A5K0UB41, YspPnk1) whose AlphaFold structural model resembles T4 Pnk. The AlphaFold (34) prediction of the C-terminal domain of YspPnk1 aligns to the N-terminal kinase domain of T4 Pnk (PDB:1ltq) with an RMSD of 2.67Å; the N-terminal domain of YspPnk1 aligns to the C-terminal phosphatase domain of T4 Pnk (PDB:1ltq) with an RMSD of 6.32Å (41) (*SI Appendix*, Fig. S5). Although YspPnk1 is predicted to be structurally similar to T4 Pnk, future studies are needed to confirm whether YspPnk1 has polynucleotide kinase activity, phosphatase activity, and whether it acts on tRNAs. With a nearly complete translational machinery, a potential T4 Pnk equivalent and YspRLIG1 encoded by the Yasminevirus genome, we speculate that a tRNA repair system may exist in Yasminevirus to protect its own tRNAs

While future studies will be required to test the existence of tRNA repair mechanisms in humans and mice, it is worth noting that mutations in the mammalian polynucleotide kinase (Clp1) also result in changes in brain tRNA and cause neurological phenotypes in both humans and mice (42–44). We speculate that Clp1, ANGEL2, and RLIG1 function together in the brain to repair tRNA. It is possible that other RNA species could also be substrates of RLIG1. Indeed, our AQ-seq experiments were biased toward small RNAs, potentially causing us to overlook changes in other RNA species. Additionally, tRNA is the most abundant RNA in the cell and can nonspecifically bind to proteins, further complicating the identification of specific RNA substrates. However, the following observations suggest that tRNA may be a physiological substrate of RLIG1. 1) The human protein shows specificity for the anticodon loop of tRNA-Lys-CTT (Fig. 2 *D* and *E*) and 2) tRNAs specifically coimmunoprecipitated with the E250A mutant of RLIG1, but not the WT protein, from HEK293A cells (Fig. 2*B*). Future work will be required to address some of these questions.

## Materials and Methods

*Materials and Methods* are included in *SI Appendix*.

### Antibodies.

Mouse α-Flag M2 antibody was obtained from MilliporeSigma (F3165). Rabbit α-Vinculin (4650S) and Lamin A/C (2032S) antibodies were obtained from Cell Signaling Technology. Mouse α-C12orf29 (RLIG1) (sc-390730) and HSP90 (sc-13119) antibodies were obtained from Santa Cruz Biotechnology. α-RtcB antibody was obtained from ThermoFisher Scientific (PA551512).

### Plasmids.

The coding sequences (CDS) of T4 Rnl1, T4 Pnk1, human RLIG1, *P. mammillata* (tunicate) RLIG1, *S. kowalevskii* (acorn worm) RLIG1, *R. bacterium* RLIG1, *C. phage* RLIG1, and *Yasminevirus sp.* GU-2018 RLIG1 homologs were synthesized as gBlocks and used directly for cloning (Integrative DNA Technologies, Coralville, IA). *Materials and Methods* for further description of plasmids used in this study.

### Bacterial Strains, Cell Lines, and Culture Media.

*E. coli* strains were grown in Luria-Bertani (LB) broth or on LB agar plates supplemented with 100 μg/mL ampicillin or 50 μg/mL kanamycin. HEK293A, Lenti-X 293 T, HeLa, and A549 cells were grown in DMEM/High Glucose medium supplemented with 10% FBS and 1% penicillin–streptomycin and incubated at 37 °C with 5% CO_2_. *Materials and Methods* for further information.

## Supplementary Material

Appendix 01 (PDF)

Dataset S01 (CSV)

Dataset S02 (CSV)

Dataset S03 (CSV)

Dataset S04 (CSV)

Dataset S05 (CSV)

Dataset S06 (CSV)

Dataset S07 (CSV)

## Data Availability

AQ-seq, TGIRT-seq and structure coordinates data have been deposited in Sequence Read Archive (SRA), PDB [PRJNA1133806 (AQ-seq) (45) and PRJNA1134178 (TGIRT-seq) (46), PDB: 9C6L (YspRLIG1) (47) and 9C6M (YspRLIG1K73M) (48)]. All other data are included in the manuscript and/or supporting information.
